# Evaluation of Stimulated Raman Histology as a Novel Method for Pathologic Examination of Urothelial Carcinoma With Unprocessed, Fresh Bladder Cancer Tissue

**DOI:** 10.1177/15330338261464295

**Published:** 2026-08-10

**Authors:** Philipp Maisch, Michael Mühlberger, Annika Beck, Wilhelm Schreen, Miles P. Mannas, Cornelia Horsch, Christian Bolenz

**Affiliations:** 1 Department of Urology and Pediatric Urology, University Hospital Ulm, Ulm, Germany; 2 Department of Pathology, University Hospital Ulm, Ulm, Germany; 3 Department of Urologic Sciences, 8166 University of British Columbia, Vancouver, British Columbia, Canada; 4 Vancouver Prostate Centre, Mohseni Institute of Urologic Sciences, Vancouver, British Columbia, Canada; 5 Institute of Epidemiology and Medical Biometry, 9189 Ulm University, Ulm, Germany

**Keywords:** urinary bladder neoplasms, bladder cancer, urothelial carcinoma, pathology, stimulated Raman histology, imaging

## Abstract

**Introduction:**

Stimulated Raman histology (SRH) is a recent microscopic technique allowing real‐time, label free, high‐resolution microscopic images of unprocessed, unsectioned tissue. Tissue samples are imaged in the operating room using a mobile SRH microscope, visualizing histology within minutes.

**Methods:**

Aim of this prospective first in human feasibility study was to evaluate the use of SRH to identify bladder cancer (BCa) and to test whether a histopathological grading between low- and high-grade tumors can be determined. Therefore, patients with suspicion of BCa, who underwent transurethral resection of bladder tumor were included. Suspicious papillary tissue was resected and subsequently scanned with an SRH microscope (NIO, Invenio Imaging Inc.). After a 10-sample training set, pathologists were tested on a 30-sample test set. Images which were initially classified wrong were re-evaluated within a consensus conference.

**Results:**

The scanning time was less than 180 seconds for all tissues samples. Individual pathologists’ overall combined accuracy (CA) for interpretation of low and high grade SRH images was 76.5 % [95% confidence interval (CI): 65.8-85.3]. After the consensus conference pathologists’ overall CA for interpretation of low and high grade SRH images was 96.3 % [95% CI: 89.6-99.2], respectively. The sensitivity for the identification of high-grade tumors was between 66.0 % and 66.7 % before the consensus conference, increasing to 100 % after pathologic consensus. The specificity of the three pathologists was between 83.3 % and 100 %. After the consensus conference, the specificity was on average 91.7 %.

**Conclusion:**

After further investigations, including the testing against benign tissue, SRH might be a reliable point of care-technique to produce high-resolution images of unprocessed bladder tumors, allowing histopathological identification of BCa. Low- and high-grade tumors can be accurately discriminated.

## 1. Introduction

Bladder cancer is the second most common malignancy in urologic cancer patients. Around 614,000 patients in 2022 were newly diagnosed with BCa worldwide.^1^ Due to demographic developments, according to the United Nations’ world population projections, more people will be affected by BCa, as this type of tumor becomes more common with increasing age.^2,3^

Assuming a growing patient population with BCa, in contrast to limited human and financial resources in patient care, the application of stimulated Raman histology (SRH) in the pathologic diagnosis of BCa could be one of many tools to handle upcoming challenges in patient care. SRH is a novel microscopic technique that enables real‐time, label free, high‐resolution microscopic images of unprocessed, unsectioned tissue. This technology holds potential to decrease the time for BCa diagnosis from days to minutes as there is no formalin fixation, hematoxylin and eosin (H&E) staining and level cutting necessary.^4,5^

**Figure 1.**
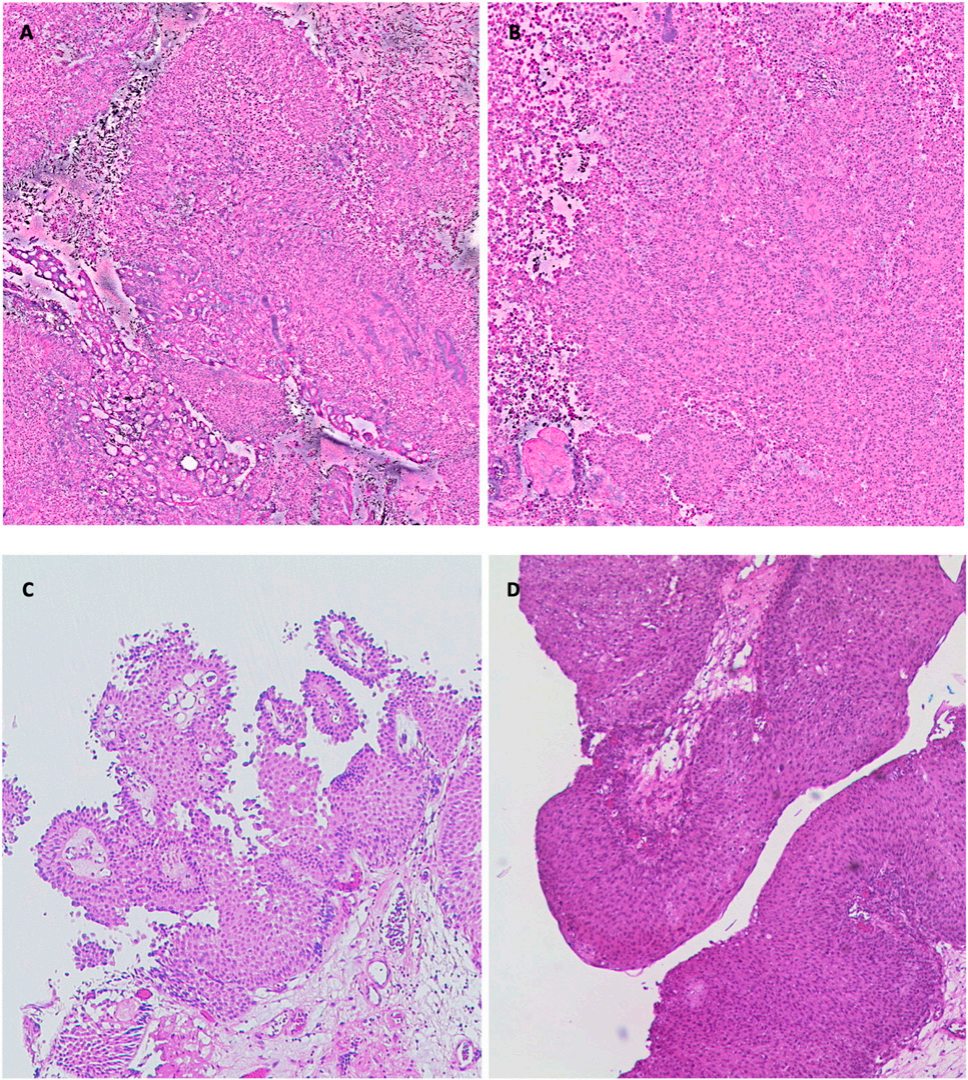
Depiction of SRH images of pTa low grade (A) and pTa high grade (B) urothelial carcinoma (magnification of 0 %) and H&E images of pTa low grade (C) and pTa high grade (D) urothelial carcinoma (50x)

**Figure 2.**
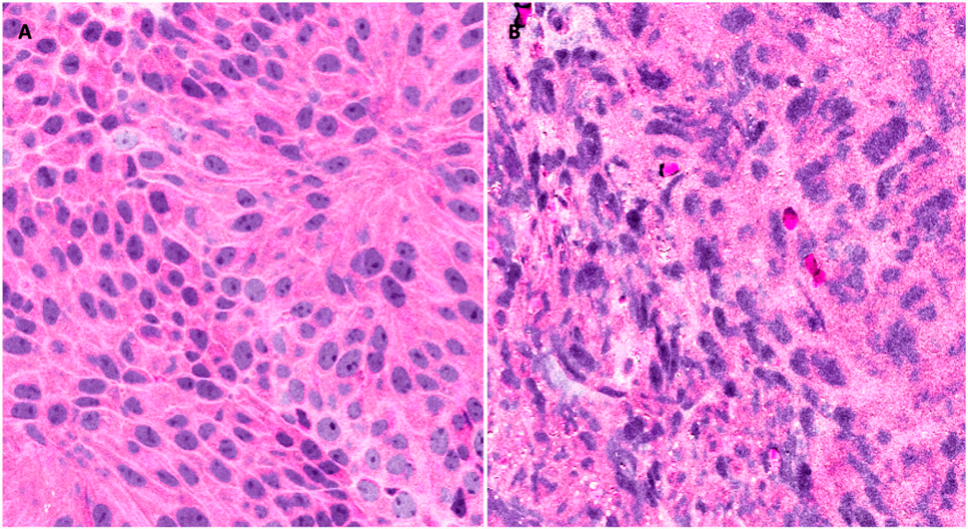
Depiction of SRH images of a pTa low grade (A) and pTa high grade (B) urothelial carcinoma with a magnification of 150 %. (A) Low-grade urothelial carcinoma: The tumor cells display a relatively uniform, homogeneous appearance with consistent cellular and nuclear morohology and balanced nuclear-to-cytoplasmic ratio. Cell borders are well-defined, and the cells are abundant in cytoplasm. The nuclei are regularly shaped, oval and contain multiple nucleoli. (B) High-grade urothelial carcinoma: The tumor cells are large and diffusely arranged, without recognizable layering. The cell borders are poorly defined. The nuclei are large, pleomorphic, and irregularly shaped, in part showing pronounced crush artifacts. Scattered inflammatory cells are also present

The workflow of SRH is straightforward and it takes limited time for image generation due to abbreviated technical setup. Tissue samples up to a size of 10 x 10 mm tissue can be imaged within 3 minutes and examined within the operating room (OR) using a mobile SRH microscope. Due to the SRHs’ pseudocoloring, the image appears like conventional H&E staining (Figures 1 and 2) and the image is transmitted to the pathologist via Wi-Fi for rapid diagnosis.

To date, already several publications describe the use of the SRH technique in neurosurgery for the diagnosis of brain tumors.^6,7^ However, this technique is under investigated in the field of urology thus far.^4,8^ Since SRH has yet not been applied in the diagnosis of BCa, the aim of this publication is to investigate feasibility for this entity. This feasibility study aims to show that, presence of BCa malignancy and histopathological grading differentiating low- and high-grade tumors can be determined using SRH images.

## 2. Patients and Methods

### 2.1. Tissue Acquisition, Preparation, and Scanning

This prospective first in human feasibility study included 24 consecutive patients with cystoscopic suspicion of BCa, who underwent transurethral resection of bladder tumor between May and August 2023 at the University Hospital Ulm, Ulm, Germany. All patients had a papillary tumor present intraoperatively, so suspicious tissue could be removed from the superficial part of the tumor. Intentionally, no biopsies were taken from the tumor base or the urinary bladder muscles to avoid compromising the pathologic staging. Histopathological staging is not an endpoint in this feasibility study. In accordance with the ethics committee’s decision for this first-in-human feasibility study, we were not permitted to resect additional healthy urothelial tissue during TURBT.

Since the removed tissue for SRH was taken from the superficial part of the tumor, additional H&E staining of this tissue was not necessary. Whether this tissue was malignant and low- or high-grade, could be determined from routine H&E histology of the remaining tissue.

The tissue collected for this study was placed in saline solution for 1 to 2 minutes to remove blood before scanning, reducing possible artifacts derived from blood.

**Figure 3.**
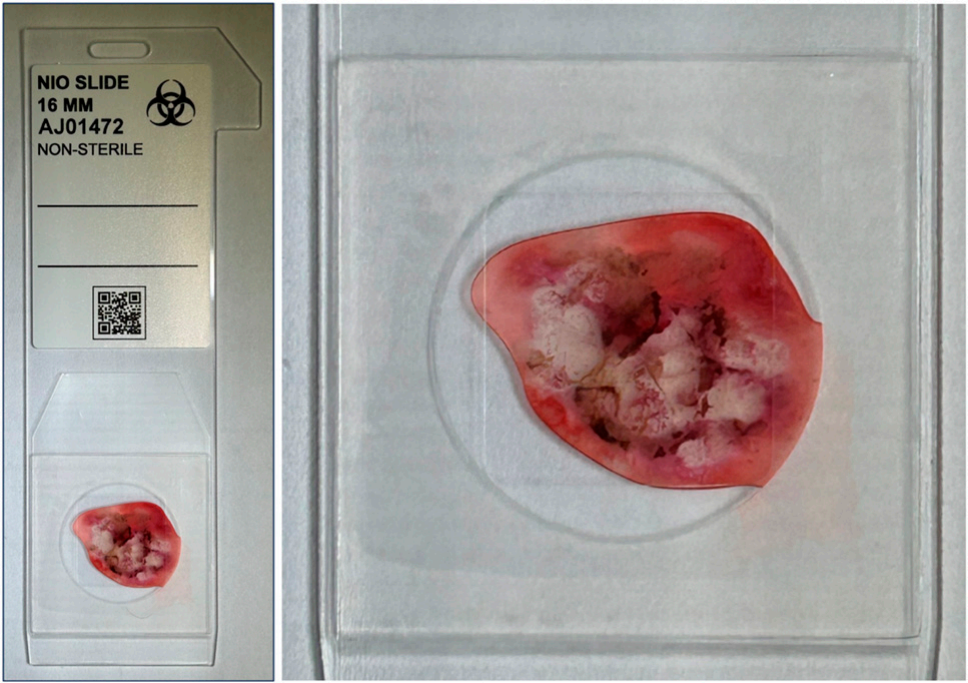
Processing of unprocessed, unsectioned papillary bladder tumor

The biopsies were placed on a SRH microscope slide and into the NIO Laser Imaging System, which is a fibre laser based stimulated Raman scattering microscope, without staining or further processing, as shown in Figure 3. All biopsies were then immediately scanned in the OR using a fibre laser based stimulated Raman scattering microscope (NIO Laser Imaging System, Invenio Imaging Inc., California, USA). The scanned tissue was not processed by routine histopathology. The images generated using the SRH microscope were made available digitally to the pathologists and evaluated using the program ImageJ, version 1.53 (National Institutes of Health, USA).

Samples with an edge length of 1-3 mm were compressed to a thickness of 230 μm on the microscope slide, a sample holder designed for use in the NIO. The thickness after compression of 230 µm was predetermined by the company’s slide. A squash preparation is required for the laser scanning process to prevent air entrapment. The 230 µm thickness provides sufficient space for a typical biopsy sample and ensures that the sample is not damaged. The acquired digital image data consisted of Raman shifts 2845 cm^-1^ and 2940 cm^-1^. 2845 cm^-1^ and 2940 cm^-1^ Raman shifts correspond to the vibrational frequencies of CH_2_ bonds, primarily lipids and CH_3_ bonds, primarily proteins and DNA, respectively. In most cases, a field of view of 5400 x 5900 pixels was chosen, resulting in a SRH image of approximately 2.5 mm x 2.8 mm. The images were generated as sequential line scans of 900 pixels width, with a pixel size of 472 nm, at an imaging depth of 10 microns below the coverslip of the microscope slide. The acquired greyscale images were automatically stitched and converted to H&E-like images.

This prospective study was approved by the independent ethics committee of the University Ulm, Ulm, Germany. All patients have given their written consent to participate in the study.

### 2.2. Pathologist Training

The ground truth for bladder tissue was made by pathologists’ interpretation of conventional H&E staining, which is defined as the accurate pathologic diagnosis.

For pathologist training a 10-sample training set of SRH images was created. This training set included 5 images of pTa low grade, 2 images of pTa high grade, 2 images of pT1 high grade and 1 image of pT2a high grade BCa (Table 1). The pathologists were aware of the corresponding histopathology of the SRH images and were thus able to familiarize themselves with the representation of cell structures using the new method of SRH. Pathologist evaluation of the SRH images was performed using ImageJ (National Institutes of Health, USA, version 1.53t).

**Table 1.** Bladder Cancer Histopathology Used for Pathologist Training and Testing

Training set		Testing set	
Histopathology	Number of images	Histopathology	Number of images
pTa low grade	5	pTa low grade	15
pTa high grade	2	pTa high grade	6
pT1 high grade	2	pT1 high grade	4
pT2a high grade	1	pT2a high grade	5

### 2.3. Pathologist Testing

Pathologic diagnosis for the BCa samples was determined based on routine pathologic examination of histopathological grading according to the World Health Organization (WHO) classification of Tumors of the Urinary System and Male Genital Organs-Part B: Prostate and Urinary Tract Tumors (Table 2).^9^ The pathologists were provided with the 30-sample test set based on histopathologic diagnosis, after completion of the training set. The BCa testing set included 30 images of urothelial carcinoma: 15 images of pTa low grade, 6 images of pTa high grade, 4 images of pT1 high grade and 5 images of pT2a high grade BCa (Table 1).

**Table 2.** Predefined Characteristics to Identify Urothelial Carcinoma, According to the 2022 World Health Organization Classification of Tumors of the Urinary System and Male Genital Organs^9^

Characteristics	Low grade	High grade
Overall view	​	Atypia in architecture and cytology
		More complex papillae than low-grade tumors
		Irregular and pleomorphic nuclei already in the overview magnification
Architecture	​	Loss of polarity/stratification disorder
Cytology	Low variation in cell nuclei size	​
Cell nuclei	Slightly enlarged cell nuclei	Significant nuclear size variance
	Little loss of nuclear polarity	Nuclear hyperchromasia
	Few cells with nuclear hyperchromasia	Irregular cell nucleus contour
		Prominent and irregular nucleoli
Mitoses	Occasional mitotic figures suprabasal	Many mitoses, some atypical
Other	​	Special forms (invasive): micropapillary, villoglandular, glandular, etc.
		Occasional anaplasia

Since all pathologists were aware of assessing images of malignant BCa, pathologists had to evaluate whether diagnosis of malignancy was possible according to SRH images. Furthermore, we evaluated the ability of pathologist to distinguish between low- and high-grade tumors based on recognizing at least 2 out of 3 characteristics of the WHO classification in the SRH images for low-grade and at least 3 out of 5 criteria for high-grade tumors. The evaluations were conducted independently by the pathologists, with no discussion or exchange regarding their assessments.

After the individual evaluation by each pathologist, all pathologists reevaluated images within a consensus conference which were initially classified wrong by at least one pathologist (no image was wrongly classified by all three pathologists). Pathologists were blinded to the histopathologic diagnosis. After a consensus classification was made, the histopathology was available within a sealed envelope. To minimize recall bias, the order of images was altered between the individual assessments and the consensus conference, with an interval of approximately four weeks between them. Since comparing this novel microscopic method with H&E staining was not an objective of this feasibility study, we did not record the time required to assess each image.

### 2.4. Statistical Analysis

All analyses were performed using the R software for statistical computing (The R Foundation, version 4.3.2). Sensitivity and specificity were computed using the caret package in R. Cohen’s Kappa was calculated to assess the concordance between two raters (R-package DescTools). Respectively the overall inter-rater reliability for all three pathologists was determined with Fleiss Kappa (R-package DescTools). Cohen’s Kappa grading concordance (κ) was interpreted as *none* for κ values between 0 - 0.20, *minimal* for 0.21 - 0.39, *weak* for 0.40 - 0.59, *moderate* for 0.60 - 0.79, strong for 0.80 - 0.90, and *almost perfect* for values greater than 0.90.^10^ As it is an adaptation of Cohen’s kappa, the same interpretation was done for Fleiss Kappa.

## 3. Results

Depending on the size of the tumor, more than one tissue sample per participant may be taken to create 2 to 12 SRH images without affecting the standard histological analysis. The scanning time was less than 180 seconds for all tissues samples. Due to low image quality of 3 samples, 27 out of 30 images were analyzed. Three images were a priori excluded by all pathologists because an assessment was not possible due to artifacts of blood.

Table 3 descriptively shows the classification frequencies of low- and high-grade tumors with SRH relative to conventional histology results for all three pathologists and the consensus conference.

**Table 3.** Classification of Bladder Tissue Using Raman Histology Relative to Conventional Histology Results

Histology results	**Individual interpretation of SRH images**							Combined accuracy (%) [95% CI]	Overall combined accuracy (%) [95% CI]
	N total	Pathologist 1		Pathologist 2		Pathologist 3			
		Correct	Incorrect	Correct	Incorrect	Correct	Incorrect		
Low grade	12	12	0	10	2	11	1	91.7 [77.5-98.3]	76.5 [65.8-85.3]
High grade	15	10	5	10	5	9	6	64.4 [48.8-78.1]	

Histology results​	**Consensus conference interpretation of SRH images**							Combined accuracy (%) [95% CI]	Overall combined accuracy (%) [95% CI]
	N total	Pathologist 1		Pathologist 2		Pathologist 3			
		Correct	Incorrect	Correct	Incorrect	Correct	Incorrect		
Low grade	12	11	1	11	1	11	1	91.7 [77.5-98.3]	96.3 [89.6-99.2]
High grade	15	15	0	15	0	15	0	100 [92.1-100.0]	

Individual pathologists’ overall combined accuracy for grading BCa SRH images was 76.5 %, [95% CI: 65.8-85.3] with combined pathologist accuracy for low grade BCa of 91.7 % 95% CI: [77.5-98.3] and 64.4 % [95% CI: 48.8-78.1] for high grade BCa (Table 3). Most frequently, pTa high grade tumors were misclassified with 4 of 6 images (Table 4). All misclassified pTa high grade tumors were classified as pTa low grade tumors. After the consensus conference pathologists’ overall combined accuracy for pathologist grading of low and high grade BCa SRH images was 96.3 % [95% CI: 89.6-99.2]. The combined accuracy for low grade images was 91.7 % [95% CI: 77.5-98.3] and 100 % [95% CI: 92.1-100.0] for high grade images (Table 3).

**Table 4.** Classification of Bladder Tissue Using Raman Histology Relative to Conventional Histology Results (for Different Grades)

Histology results	SRH interpretation				
	N total	Pathologist 1	Pathologist 2	Pathologist 3	Consensus conference
		Misclassification	Misclassification	Misclassification	Misclassification
pTa, low grade	12	0	2	1	1
pTa, high grade	6	4	4	4	0
pT1, high grade	4	0	0	0	0
pT2a, high grade	5	1	1	2	0

The sensitivity for the identification of high-grade tumors was between 66.0 % and 66.7 % before the consensus conference; this was increased to 100 % with the consensus conference. The specificity of the three pathologists was between 83.3 % and 100 %. The consensus conference resulted in a specificity of 91.7 % for identification of high-grade tumors with SRH images (Table 5).

**Table 5.** Pathologists’ Sensitivity and Specificity for Identification of High-Grade Tumors

​	Sensitivity (%) [95% CI]	Specificity (%) [95% CI]
Pathologist 1	66.7 [38.4-88.2]	100.0 [73.5-100.0]
Pathologist 2	66.7 [38.4-88.2]	83.3 [51.6-97.9]
Pathologist 3	60.0 [32.3-83.7]	91.7 [61.5-99.8]
Pathologist consensus conference	100.0 [92.1-100.0]	91.7 [77.5-98.2]

The Cohen’s Kappa grading concordance (κ) was strong, with a range of 0.841 to 0.847 (Table 6). Also, the calculated inter-rater reliability of 0.845 for all pathologists, calculated with Fleiss Kappa, was strong.

**Table 6.** Cohen’s Kappa for Inter-rater Reliability

​	Grading concordance (k) Cohen’s kappa [95% CI]		
	Pathologist 1	Pathologist 2	Pathologist 3
Pathologist 1	-	0.847 [0.646-1.049]	0.841 [0.630-1.053]
Pathologist 2	0.847 [0.646-1.049]	-	0.847 [0.646-1.049]
Pathologist 3	0.841 [0.630-1.053]	0.847 [0.646-1.049]	-

## 4. Discussion

The aim of this study was to assess the feasibility of SRH to be utilized for rapid histopathological diagnosis of BCa. Furthermore, it was our intent to demonstrate that this new method can help pathologists distinguish between low and high grade tumors.

By means of this study we demonstrated that SRH can be used in the pathological assessment of BCa. Using the SRH technique it was possible to reproducibly induce label free images of fresh BCa tissue for pathologists to identify BCa. After excluding three images with poor image quality, all SRH images could be assessed by the pathologists. Even within the framework of a standard histopathological assessment using H&E staining, it may happen that tissue cannot be assessed due to artifacts. We therefore consider the exclusion of three SRH images to be tolerable and consider the SRH technique capable of being used in the routine histopathological assessment of BCa providing sufficient images. Although we only removed the superficial part of the tumor to avoid affecting routine histopathological assessment, we were able to generate at least two SRH images of unprocessed BCa tissue. If the entire tumor material would be examined, sufficient images would be available for the routine assessment.

Furthermore, this study shows that SRH images of fresh tissue can be accurately interpreted by pathologists. In order to familiarize the pathologists with this new technique, a training set of 10 SRH images was provided. After evaluation of this training set, the individual pathologists’ assessment of the testing set showed a moderate overall combined accuracy of 76.5 % [95% CI: 65.8-85.3]. In this phase of the individual assessment, a very good combined accuracy of 91.7 % [95% CI: 77.5-98.3] for low grade tumors and a moderate combined accuracy of 64.4 % [95% CI: 48.8-78.1] for high grade tumors was already achieved.

Although more high grade (4 to 6) than low grade (1 to 2) tumors were misinterpreted during the individual pathologists’ interpretation of the SRH images, this discrepancy was eliminated by consensus conference. After the consensus conference, the overall combined accuracy was very good with 96.3 % [95% CI: 89.6-99.2]. The combined accuracy for low-grade and high-grade carcinomas was increased to 91.7 % [95% CI: 77.5-98.3] and 100 % [95% CI: 92.1-100.0], respectively. Within the consensus conference, pathologists identified in images initially classified as low grade carcinomas, focal areas showing increased variability in nuclear size and irregular (non-rounded) nuclear contours (see Table 2, WHO classification, cell nuclei). Although these focal areas predominantly composed of low-grade dysplasia, pathologists became aware of high-grade dysplasia within a heterogeneous dysplasia pattern and then classified these images correctly as high grade carcinomas. Since the consensus conference has clearly improved the accuracy of pathologists’ interpretation, it seems that a certain learning curve and collegial exchange is required to become familiar with interpretation of BCa with this new technology. As with new methods of interpretation, learning curves can be variable; however, we assume, that with a more further training the pathologists’ performance will improve. For further implementation of SRH as a routine diagnostic tool, user may benefit of a guided training before applying SRH clinically, so that this new method requires the same human resources than diagnosis for H&E slides. The result of the consensus conference should be interpreted in a differentiated manner, as it was developed within the framework of a discussion and reflects the opinions of all participating pathologists.

Due to a maximum scanning time of 180 seconds per tissue sample, the histopathological processing of bladder specimen via SRH could have a direct impact on the intra- and postoperative course of patients undergoing cystectomy or TURBT. After the assessment by the pathologist, the malignancy of suspicious tissue could be confirmed within minutes, allowing immediate postoperative instillation of chemotherapeutics to be used more specifically. In addition, the indication for a necessary re-resection in case of first diagnosis of pTa or pT1 high-risk BCa could be made immediately, which might reduce the organizational effort for upcoming surgery. For locally advanced BCa, the SRH technique could be used as an intraoperative frozen section during cystectomy. In locally advanced findings it could be possible to contribute to an R0 resection, using SRH imaging in addition to the usual frozen sections of the ureters and urethra.

It has been shown that the time between diagnostic procedure and diagnosis is the most anxiety provoking during a patient’s cancer diagnosis and treatment.^11^ Ultimately, an immediate diagnosis spares the affected patient an agonizing waiting period associated with high anxiety levels, in which confirmation of a malignant tumor is pending and a potential further therapy is unclear.

However, the above-mentioned aim of this study, as well as its’ implementation, results in limitations of the presented results. In this study, only SRH images of malignant findings were interpreted. The pathologists were aware of this and no decision had to be made as to whether the tissue was malignant or benign. Due to the ethics committee vote on this first in human feasibility study, we were unable to resect additional healthy urothelial tissue during TURBT. Of course, further investigations should demonstrate, that with the use of SRH imaging benign and malignant bladder tissue can be differentiated by pathologists. Those further investigations have to include also more patients. The limited sample size of 30 images is due to the nature of a feasibility study.

Comparing SRH with the established H&E staining method, both methods can generate sufficient images or slides from the available tissue to adequately analyze it. Due to the comparable representation of the tissue by SRH, the methods of assessment are comparable for pathologists. In these respects, both methods are equivalent. However, we see an advantage for SRH, as for visualization no cutting or staining is necessary. This allows more tissue to be examined in a shorter time. However, implementing SRH is associated with the acquisition costs for an SRH microscope. These costs can be shared with other departments, as the SRH microscope is a mobile device and can therefore be used in various areas. In contrast to H&E staining, which is available worldwide, the SRH method is not yet established. According to the learning curve shown in this study, training of pathologists is necessary, which entails a significant investment of time and personnel.

The effect of SRH on standard BCa histopathologic analysis has yet been determined; therefore, in order not to influence routine histopathology analysis we obtained tissue from papillary tumors superficial to the tumor base. Within this study, it was therefore not possible to assess whether BCa was invasive. However, since the tissue samples used for SRH imaging can also processed for H&E staining, the ability to determine the presence of invasion and muscle invasive disease may also be assessed using SRH in future studies.

In the field of neurosurgery, where SRH has been in use for some time and is more widely established, initial studies have already demonstrated the successful application of artificial intelligence (AI) in the analysis of images generated with SRH.^12-15^ Thus, AI-based diagnostic approaches could potentially complement existing laboratory techniques, thereby enhancing both the accessibility and the speed of molecular diagnostics in comprehensive patient care, pushing the impact on SRH imaging even further.^14^

In summary, this study shows that in the histopathological assessment of BCa the application of the SRH technique is a promising approach.

## 5. Conclusion

The SRH technique is a reliable method that can produce images of unprocessed bladder tumor tissue for histopathological evaluation in less than three minutes. Due to high-resolution images, it is possible, to identify bladder cancer and to differentiate between low- and high-grade tumors. Further evaluation of this new technique may lead to clinical implementation of this method to modify the diagnostic and treatment algorithm of BCa.

## Data Availability

All data generated or analyzed during this study are included in this published article.*

## Appendix

### Abbreviations

BCa: Bladder cancer
CI: Confidence interval
CA: Combined accuracy
CH2: Methylene group
CH3: Methylene group
DNA: deoxyribonucleic acid
H&E: Hematoxylin and eosin
OR: Operating room
SRH: Stimulated Raman histology
USA: United States of America
WHO: World Health Organization.
